# The Motivations of Citizens to Attend an eHealth Course in the Public Library: Qualitative Interview Study

**DOI:** 10.2196/60612

**Published:** 2025-04-28

**Authors:** Lucille Standaar, Adriana Margje Israel, Rosalie van der Vaart, Brigitta Keij, Frank J van Lenthe, Roland Friele, Mariëlle A Beenackers, Lilian Huibertina Davida van Tuyl

**Affiliations:** 1 Centre for Public Health, Healthcare and Society, Department of National Public Health and Healthcare, National Institute for Public Health and the Environment, Postbus 1, Bilthoven, 3720 BA, The Netherlands, 31 631135143; 2 Department of Tranzo, School of Social and Behavioral Sciences, Tilburg University, Tilburg, The Netherlands; 3 Department of Public Health, Erasmus MC, University Medical Center Rotterdam, Rotterdam, The Netherlands; 4 Research Group Technology for Healthcare, The Hague University of Applied Sciences, The Hague, The Netherlands; 5 Netherlands Institute for Health Services Research, Utrecht, The Netherlands

**Keywords:** digital health, help-seeking behavior, socioeconomic factors, community health services, older people, eHealth, older adults, geriatric, support, eHealth literacy, interview, Netherlands, thematic data analysis, health literacy, mobile phone

## Abstract

**Background:**

There is worldwide recognition of the potential increase of digital health inequity due to the increased digitalization of health care systems. Digital health skill development may prevent disparities in eHealth access and use. In the Dutch context, the public library has started to facilitate support in digital health skill development by offering public eHealth courses. Understanding the motivations of people to seek support may help to further develop this type of public service.

**Objective:**

This is a qualitative study on the motivations of citizens participating in an eHealth course offered by public libraries. The study aimed to explore why citizens were motivated to seek nonformal support for eHealth use.

**Methods:**

A total of 20 semistructured interviews with participants who participated in an eHealth course were conducted in 7 public libraries across the Netherlands. The interviews were conducted between April and June 2022. Purposive sampling took place in the public library during the eHealth course. The interviews covered participants’ motivations, attitudes, and experiences with eHealth use and their motivations to seek help with eHealth use. Interviews were audio-recorded and transcribed. Themes were identified via a comprehensive thematic data analysis.

**Results:**

The participants were 51 to 82 years of age (average 73.5, SD 6.6 y) and 14 (70%) participants were female. Three motivational themes were identified: (1) adapting to an increasingly digital society, (2) sense of urgency facilitated by prior experience in health care, and (3) a need for self-reliance and autonomy. Additionally, participants expressed a general desire for social contact and lifelong learning. A lack of adequate informal support by friends and family for digital skills and positive experiences with formal support from public libraries stimulated the participants to seek formal support for eHealth use.

**Conclusions:**

We show that the participants had a feeling of urgency that sparked their motivation to seek nonformal support in the public library. Motivations to participate in the eHealth course stemmed from the need to adapt to the digital society, being a patient or a caregiver, or the need or wish to be independent from others. Participants of the study were mainly older female adults who had native language abilities, up-to-date digital devices, and time. It is likely that other populations experience similar feelings of urgency but have other support needs. Future research should explore the needs and attitudes of nonusers and other users of digital health toward seeking support in eHealth access and use.

## Introduction

With the growing demand for health care services, eHealth is increasingly recognized as a critical tool for efficiently managing and delivering health care [1]. Through the adoption of patient portals, telemonitoring tools, and smartphone apps, patients can experience improved access to health information, shared decision-making, and self-management [2,3]. However, not everyone within society is able to access and use digital health care [4-12]. The increased implementation of digital tools in health services is linked to the widening disparities in health [13].

The need for more emphasis on equity in the access and use of digital health care systems is recognized worldwide. Research shows that disparities in the use of digital health are common in member states of the European Union [14,15]. The causes of digital health inequities derive from unequal access to resources, language, and digital health skills required for effective eHealth utilization [8-10,16-22]. World Health Organization and Organization for Economic Co-operation and Development encourage countries to adopt digital health literacy policies on citizen and health system levels [23,24]. Yet, only 27 out of 52 European Union member states have developed policies on digital health literacy (2023) [23]. Digital health literacy is defined by Norman and Skinner [25] as “the ability to seek, find, understand, and appraise health information from electronic sources and apply the knowledge gained to addressing or solving a health problem.” Enhancement of digital health skills is one of the avenues for achieving a more equitable digital health care system [23,24,26].

In The Netherlands, the COVID-19 pandemic created awareness about the lack of digital health literacy which resulted in a collaborative effort between the government and the public library organization to facilitate support and education in the development of digital skills, including digital health skills [27-29]. As a result of this collaboration, the public library in the Netherlands has evolved into an organization that aims to support “those who need help” [30,31]. The public library offers a nonformal free-of-charge eHealth course, designed to enhance digital health skills [31]. In the literature, 3 categories of support are defined; formal support: institutionalized education with officially recognized awards, nonformal support: institutionalized support and education without officially recognized awards, and informal support: support via social networks [32]. Support via the public library is a form of nonformal support. As the public library is freely accessible to the whole population, costs or resources would not deter the use of this type of support. However, the first step to participate remains to be taken by the individual and depends on their motivation to improve their digital health skills [17,19,31].

A better understanding of the motivations of people who seek nonformal support can create useful insights into the sentiments that drive eHealth adoption and support-seeking behavior. These insights can be used to improve eHealth implementation and the design of eHealth support strategies. This study aims to explore the motivations that drive participants in a freely accessible eHealth course to seek nonformal support for eHealth use. Building on our prior research and other findings, which demonstrated that education was associated with motivation to use eHealth [18], this study’s findings will also be explored through the lens of the educational background of people who seek support.

## Methods

### Study Design

This study aims to understand and explore the motivations of participants to seek support with digital health skill development. A qualitative study design with semistructured interviews was deemed most appropriate as this study aims to explore and describe the motivations to seek help in the eHealth use of participants of an eHealth course [33]. Due to the explorative nature of the research question, this study follows the interpretivism approach [34]. As this study has an explorative nature with the aim to create a deep understanding of the motivations of the participants to seek support, the results of this study will be unique to the study setting and the study sample. This paper adhered to the SRQR (Standards for Reporting Qualitative Research) checklist (Checklist 1) [35].

### Ethical Considerations

The study was declared to fall outside the scope of the Dutch Medical Research Involving Human Subjects Act by the Clinical Expertise Center of the Dutch National Institute for Public Health and the Environment (VPZ-559). Transcripts of the interviews and the informed consent forms are stored and protected at the Dutch National Institute of Public Health and the Environment. All participants provided their informed consent prior to conducting the interviews. Participants received a 15€ (US $16.047) voucher for their participation. Transcripts were deidentified.

### Setting

Digital health care is widely implemented within the Dutch health care system and involves data storage, interaction between (health care) professionals, interaction between health care professionals and patients, and freely accessible health information websites and apps [36]. Implementation of digital health care has been experimented with over the last decades within the Dutch health care system and is stimulated by the government [37-39]. The Netherlands is one of the best-connected countries in the European Union, with 98% of its citizens connected to a type of internet connection [40]. The history of experimentation with eHealth implementation, governmental support, and the wide availability of the internet is likely to support the digitalization of the health care system further.

The eHealth course “DigiVitaler” is part of the web-based educational material from the National Public Library and available to all public libraries since 2021 [41]. In 2022, 60 out of 137 public libraries offered the eHealth course [41]. This course is structured into multiple classes, each covering a specific eHealth topic, such as the patient portal, video consultations, and web-based health information. Participants are introduced to eHealth by providing hands-on experience with eHealth apps in simulated digital environments. The course consists of 1 up to 8 classes, each with a duration of 2‐3 hours, and accommodation of 2‐12 participants. Public libraries are free to choose the number of classes they offer. Public libraries used printed, word-of-mouth, and social media advertisement strategies to announce the course, disseminated via libraries’ own platforms, local newspapers, and local organizations.

### Recruitment

Purposive sampling of participants who had experience with the eHealth course was performed by 3 researchers (LS, AMI, and Anita Suijkerbuijk) in the context of 7 public libraries. Researchers aimed to recruit a representative sample of all participants of the eHealth course. Through contact with the developer (Stichting DigiSterker), the researchers retrieved contact information of the libraries offering the eHealth course. The course developer was not involved in this study. Libraries were contacted via email and received information about the study with the request to allow the researchers to be present during the eHealth course for participant recruitment. A total of 7 out of the 26 contacted libraries agreed to participate. Reasons for libraries to decline were course cancellations due to a low number of course participants, no course dates in the data collection period, no response, or unwillingness to participate in the study. After obtaining consent from the librarians, the researchers (LS, AMI, and Anita Suijkerbuijk) attended 9 classes in 7 libraries located in rural and urban areas in the Netherlands. At the start of each class, potential participants were introduced to the study through a presentation and a flyer. With the approval of the course trainer, researchers would play an active role during the class, providing support and answering the course participants’ questions about the course material. At the end of the class, participants were invited for an interview. Participants who expressed interest were provided with detailed study information, a consent form, and the opportunity to ask questions. Recruitment and interview procedures were designed to limit the participants’ potential efforts to participate in the study. Procedures were conducted in a familiar context for the participants and required limited travel time and no digital skills. All participants were interviewed at the library, either immediately following the course or at a preferred later date. Telephone interviews were an option but were not requested by the participants. Digital interview formats were not considered as it was highly likely that the participants had limited digital skills, which would potentially pose a barrier to recruitment.

### Participants

Participants were eligible to participate if they were older than 18 years of age and participated in the eHealth course. Educational background was categorized according to the International Standard Classification of Education (ISCED), where a low educational background is categorized as ISCED 0‐2, intermediate as ISCED 3‐4, and high as ISCED 5‐8 [42]. The rurality of the participants’ living area was derived from the area where libraries were situated. Rurality was determined via the rurality classification from Statistics Netherlands [43]. A total of 48 participants attended the classes visited by the researchers, of which 20 (20/48, 42%) participants participated in the study. Reasons for nonparticipation were lack of time or interest or the participant’s own perception of lack of experience with eHealth.

### Data Collection

In this study, motivations related to seeking help and the use of eHealth were studied. The themes of the interview guide covered: the demographics of the participant; the motivations, self-efficacy, and attitudes for eHealth use; motivations regarding seeking and receiving support for eHealth use; and social context of support and eHealth use. The interview guide can be found in Multimedia Appendix 1.

Multiple theories describe the transition from motivation to behavior [44-46]. The 3 concepts, attitude, social norms, and self-efficacy, are often described to result in motivation [44-46]. In this study, these theories were used as a means to develop the interview guide that covers the main concepts of motivation. Attitude refers to positive and negative beliefs about the consequences of certain behaviors [44-46]. Social norms include the beliefs of others about the behavior [44-46]. Self-efficacy expectations refer to the beliefs of one’s personal capacities to actually change behavior [44-46]. Questions that cover these concepts entailed questions such as: “What or who caused you to seek support with eHealth use and why?” (attitude and social norm); “What was the role of family; friends; health care professionals; the library; or the municipality to use eHealth?” (social norm); “Would you be able to use eHealth yourself after the course?” (self-efficacy). The interview guide was developed by LS and AMI and reviewed by LT, AS, and MAB.

Semistructured interviews were conducted face-to-face by researchers LS, Anita Suijkerbuijk, and AMI, all experienced in conducting qualitative research. None of the researchers knew the participants prior to this study. The data collection period lasted from April until June 2022. All interviews were audio-recorded with participants’ permission and lasted 30‐60 minutes. After each cycle of 6 interviews, the content of the interviews was discussed by LS and AMI. After 20 interviews, no new information regarding motivation to seek help with eHealth use was heard. At this point, it was concluded that data saturation was reached.

### Data Analysis

Deidentified verbatim transcriptions of the interviews were analyzed with the “Codebook Thematic Analysis” approach [47]. This approach allows for a structured process of coding, theme development, and conceptualization in a multidisciplinary team while maintaining the open, inductive approach of reflective thematic analysis to explore the motivations of the participants in-depth [48]. First, transcripts were read and summarized for data familiarization. A deductive codebook was derived from the interview guide. The first 2 interviews were double-coded by LS, AS, LT, and AMI, who then collaboratively defined and fine-tuned the codebook. After consensus, the remaining interviews were coded and cross-checked by LS and AMI, using MaxQDA (VERBI Software) for data management. This deductive analysis was performed to create an overview of the data gathered, followed by an inductive analysis to identify underlying patterns. The following topics were analyzed inductively: motivation for eHealth use, motivation for seeking support in eHealth use, and motivation to participate in the eHealth course. The first 20% of the transcript segments per topic were inductively coded by LS and AMI separately. After reaching a consensus, the rest of the transcripts were coded and cross-checked by LS and AMI. The inductive codes were arranged into candidate themes. Candidate themes were then further defined through discussion between all researchers to determine the final themes. After analysis, all the transcript summaries were reread and the presence of the themes for every participant was evaluated through the lens of their life course with the aim of identifying possible differences according to educational background. Quotes were translated using the translation software DeepL (DeepL SE) and these were checked by a native English speaker with limited Dutch fluency.

## Results

### Overview

A total of 5 men and 15 women with an average age of 73.5 (SD 6.6; range: 51‐82) years participated in this study. All participants were White and fluent in the Dutch language. Most participants (n=13) lived in an urban area. Seven participants were categorized with a low educational background (LE), 6 participants with an intermediate (IE), and 7 participants with a high educational background (HE). All participants made use of 1 up to 4 digital devices and 14 participants had prior experience with digital health services. The study sample’s (n=20) demographic overview can be found in Multimedia Appendix 2. Participants mentioned three types of support to develop their digital skills: (1) family, friends, and acquaintances; (2) the public library and other public or social organizations; and (3) commercial resources. Participants were either introduced to the eHealth course by library staff during participation in other courses offered by the library, advertisements in the local newspaper, or via their church, social organization, or computer club.

### General Experience With the Digitalizing Society

The participants’ experiences with and attitudes toward the digital world varied, yet all expressed some degree of difficulty using digital devices and services due to a perceived lack of adequate digital skills. Many pointed out that it is difficult for “their generation” to keep up with the latest developments within an ever-changing field. “Their generation” pertains to those who did not grow up with computers or the internet and only learned this later in life, even though some had multiple years of digital experience.

*I think it’s a shame that there’s no choice anymore. There’s obviously a generation of us that needs to have a transition that’s a little bit smooth, and not like: bam, next year everything’s just different. I think a lot of people my age have problems with that.*

[Participant 5, female, 72 years of age*,* LE]

For most, this digital experience was work-related where they were trained in specific, mostly simple computer programs useful for their jobs. However, this was different from their experience in this day and age where every aspect of life has become infused with digitalization and there often is little to no alternative left.

### Themes

#### Overview

Three themes were identified to describe individuals’ motivations to seek help for eHealth use: (1) adapting to an increasingly digital society, (2) a sense of urgency facilitated by health care experience, and (3) a need for self-reliance and autonomy. Additionally, a desire for social contact and lifelong learning were general reasons to participate in courses offered by the public library. An overview of the themes, the topics, and the number of codes linked to a topic is presented in Multimedia Appendix 3.

#### Theme 1—Adapting to an Increasingly Digital Society

##### Overview

The majority of the participants experienced that society is becoming more digital and they feel the urgency to try and keep up with the digital transition to be able to function as participating citizens. Three aspects encompass this motivation: (1) realizing the lack of one’s own digital skills, (2) feeling forced by society to keep up with digitalization, and (3) a desire to feel included in society and be prepared for the future. This theme was identified among all participants, regardless of educational level. It seems in this respect that among the participants of this course, their age and the fact they have little experience with digital technology played a bigger role than their educational background.

##### A Lack of Digital Skills

The experience and expectation of lacking digital skills created a sense of urgency for most participants to learn how to use eHealth. Participants found it uneasy to navigate through the digital world in general. Many participants reported that developing and maintaining digital skills requires continuous effort.

*But it’s my experience, if you don’t do it regularly, it disappears again. Then you think, “Oh dear, how did I do this again?” And just figuring that out, it’s very frustrating.*

[Participant 11, female, 73 years of age, HE]

Some participants experienced fear of using the computer since they were afraid to make (irreversible) mistakes.

*The fear of buttons for me is that I think I’m pressing such wrong buttons that everything’ll disappear.*

[Participant 15, female, 74 years of age*,* HE]

Some participants tried to use eHealth but failed due to a lack of digital skills. Others found that their lack of digital skills limited them in using eHealth to its full potential or impeded their preferred option in health care services. This motivated them to start learning.

##### Feeling Forced by Society to Keep Up With Digitalization

As services and goods become increasingly digitalized without alternatives, participants felt compelled to adapt and commit to them.

*Then I think: something is being shoved down my throat again. I don’t want that at all. But still, I get it: you have to, whether you want to or not.*

[Participant 19, female*,* 74 years of age*,* LE]

Anxiety about falling behind and becoming isolated from society or losing part of their autonomy was common. Feelings of insecurity, shame, or feeling scared using new digital technology were no exception, as previous experiences with or news warnings about scamming or loss of privacy raised their concerns.

*A copy of my passport, I find that very uncomfortable when asked for it. […] I don’t really dare to [upload] ... because I’m afraid, yes it’s very farfetched but it does happen. I’m still afraid of being taken over by cybercriminals.*

[Participant 17 (female, 51 years of age, HE]

Many participants foresaw that health care services would further digitalize. They feared diminished access to care if they were unable to use eHealth, which resulted in beliefs that they needed to forcefully commit. However, the barriers participants experienced for the use of eHealth were numerous. Negative experiences contributed to the reluctance participants felt to use eHealth: lack of uniformity between eHealth apps, difficulty identifying appropriate health information, and difficulty in phrasing health problems in digital form.

*Too complicated for us at this age [asking questions via e-consultation]. You have to do too many steps. You have to log in, you have to type, you have to explain what it is, and everything has to be short.*

[Participant 10, female, 78 years of age*,* HE]

##### Desire to Feel Included in Society and Be Prepared for the Future

The desire to keep up with digital developments led to an intrinsic motivation to learn how to use eHealth. The participants felt that by keeping up, they stay part of society and are prepared for possible (future) encounters with eHealth. Some participants felt the urgency to learn how to use eHealth now because they foresaw that this would become harder as they age.

*It is the future where we are headed, soon you have to do a lot by computer and by smartphone. Then it’s really nice if I know how to use it, because if I’m approaching 70 and I still have to learn, well then it is too late.*

[Participant 6, female*,* 66 years of age*,* LE]

These participants noticed that they were the early adopters of eHealth in their social network. Friends and relatives did not share the same interests or understand why the participant was willing to learn digital skills to use eHealth. This is part of the reason why nonformal support in the form of the eHealth course is a particularly attractive option, as the informal network of the participants is not as knowledgeable.

### Theme 2—Sense of Urgency Facilitated by Prior Health Care Experience

#### Overview

The second theme depicts that the participants felt motivated to seek help to use eHealth through their prior health care experience. Three aspects encompass this theme: (1) the responsibility as a patient or caregiver, (2) experience with the benefits of eHealth, and (3) the attitude of health care professionals toward eHealth. This theme was mentioned by all participants from the low-educated group, a few participants in the intermediate-education group, and half of the high-educated group. Regardless of their educational background, the participants who have had experience in the health care system felt more urgency to use eHealth.

#### The Responsibility as a Patient or Caregiver

Many participants had some form of experience with health care, although not all with eHealth. Being a patient or caregiver to their partner or child stimulated a feeling of responsibility to take an active role in the care process. For these participants, this active role encompassed the ability to use eHealth. Participants illustrated this with examples of preparing hospital appointments or looking up information in their electronic patient records. They are able to take more time to think about the medical information before a consultation to prepare questions, and also afterward to reread the information given during a consultation. eHealth allowed participants to better process the information and use this to their advantage.

*My husband was in the hospital with a heart attack [...] and I saw everything was digital there. I had already looked up his medical history once and printed it out because my husband forgets a lot. [...] And I think, now they are going to start something else [a new digital system] in the hospital again. I have to learn this from the start, and I think my husband has to go along with that.*

[Participant 13, female*,* 70 years of age*,* LE]

A few participants expressed that they feel this type of active role is expected of them by medical professionals to be able to partake in shared decision-making. These experiences motivated the participants to learn more about eHealth when they were presented with the opportunity.

#### Experience With the Benefits of eHealth

Prior experience with health care created perceptions that eHealth can be beneficial and can be a necessity in health care. The benefits participants mentioned were less travel time, the opportunity for digital health care appointments at home, improved exchange of health information between health professionals and organizations, and an improved overview and insight into their personal health information.

*The next time I visit my doctor and I need information, then beforehand I’ll read [my file]. [...] I’d rather know what’s on the record about me first. Maybe I can ask another sensible question. Because otherwise you’re just a clueless patient.*

[Participant 14, male, 76 years of age*,* IE]

Some participants also believed that eHealth could contribute to the efficiency of the health care system, which they deemed positive for themselves and society. Most participants were not familiar with the possibilities of eHealth before their health care experience. After experiencing these benefits, participants felt eager to learn about the possibilities of digital care. As a result, they sought nonformal support.

#### Attitude of Health Care Professionals Toward eHealth

A positive attitude toward eHealth or regular use of eHealth by health professionals motivated participants to use eHealth and learn skills that enable eHealth use. Some participants decided to do the course after a health professional suggested or used eHealth for their care.

*Yes, Thuisarts.nl [a website that provides easily readable patient information on primary health care issues, issued by the Dutch College of General Practitioners], I consult that often. [...] That’s what I agreed with the general practitioner at the time. He said, “Did you look on Google again?” [...] And then, “If you go to Thuisarts.nl, that’s very reliable.” And I just do that.*

[Participant 12, female*,* 80 years of age IE]

Most participants indicated they never received any information about the possibilities of eHealth via their general practitioner or other health care professionals. Some participants argued that better promotion of eHealth is needed to spread awareness and stimulate use.

### Theme 3—Need for Self-Reliance and Autonomy

#### Overview

Participants’ motivation to seek help for eHealth use was also tied to their wish for independence. The need for self-reliance and autonomy was described by three aspects: (1) limited informal support, (2) discomfort with dependency on others, and (3) discomfort with loss of their own autonomy. This theme was identified equally among all participants, regardless of educational level. Thus, again, the social circumstances such as having children and the existence or absence of a social network seemed to affect the participants more directly than their educational background.

#### Limited Informal Support

Limited or inadequate informal support resulted in participants feeling forced to become independent users of eHealth. In a few cases, limited support meant that participants were childless, whereas they felt that society often wrongly assumed that their family would take care of them. This can create a sense of loneliness, but also motivation to learn these skills.

*I do feel more or less obliged [to become independent in eHealth use]. [...] because I have nobody to fall back on. Everyone says: oh, you just ask your children. Well, I don’t have any.*

[Participant 1, female*,* 73 years of age, HE]

Other participants expressed they never felt a need to learn because their spouse would take care of all things digital. However, after their partner falls ill or has passed away, they are on their own and feel they have no other choice but to learn these skills themselves. In addition to the absence of informal support, some participants also experienced inadequate informal support, when the support they receive does not match their needs and wishes. This is especially true for the help the participants receive from their children or grandchildren, who are digital natives, and proceed to quickly resolve any issues. This is often done in such a way that the participants are left clueless about what the solution entails and are not stimulated to learn how to tackle these problems themselves. They continue to be dependent on others, which frustrates them. To be able to develop these skills on their own, they feel they need more structured explanations, time to practice, and patient teachers which the course offers them.

*Otherwise, I always have to ask one of the grandchildren. […] They’ll help me, but it’s all quick, quick, quick. Well, that doesn’t work. […] That’s why I’m here taking this course.*

[Participant 6, female, 66 years of age, LE]

#### Discomfort With Dependency on Others

To lean on others for help in eHealth use was uncomfortable for some participants due to their desire for independence, leading to their wish for independent eHealth use. Usually requesting help with small or acute matters within close ties such as family is acceptable, but depending on others outside of the family such as neighbors or friends feels uncomfortable and like they are burdening others. Some participants expressed that even asking family for help can feel like they are asking too much of their children, especially if they feel their children are busy with their own household, young children to take care of, or demanding jobs. The course offers a welcome alternative, where the participants do not feel like a burden.

*Because I wanted to deal with it [eHealth] more easily. I do have children who want to help, but everyone still has their own thing. I don’t want to become dependent on my kids.*

[Participant 10, female*,* 78 years of age, HE]

Some also reported that they are afraid or find it difficult to ask for help from formal institutions such as the municipality. This, in turn, also has to do with a lack of trust and safety. Participants feel discouraged to ask for support from these types of organizations because they think they will not get sufficient guidance. Participants found commercial services to be too expensive, incompatible with their own equipment, insufficiently informative, or too time-consuming.

#### Discomfort With the Loss of Their Own Autonomy

Asking for help or being unable to use eHealth meant for some participants that they could not manage their own life and health. Their discomfort lies in their dependency on others rather than being uncomfortable burdening others with their need for help. It means the loss of autonomy which many of the participants experienced their whole lives through their employment, role in the household, and management of daily life. But as they get older, they feel they lose this freedom as it becomes harder to keep up with digital innovation. Many participants feel they want to be able to solve problems on their own, without asking for help. Attending the course feel like they are in control and actively solving the problem by learning new skills and by doing something themselves. They experience this as more empowering than asking someone else to solve their problems for them.

*Of course, I always wanted to do everything myself. Now you have to [ask for help] every time... That’s the thing. Every time you have to give up a piece of your personality. That’s how it feels.*

[Participant 19, female*,* 74 years of age*,* LE]

In addition to these 3 themes related specifically to the motivation for eHealth use support, the participants expressed a general desire for social contact and life-long learning as a reason to participate in the eHealth course. With regards to social contact, participants reported finding solace and support to tackle digital (health) skills together with likeminded people, where they are not the only ones experiencing difficulty with new technology and have the opportunity to learn from each other’s questions and mistakes without feeling like the odd one out. This feeling of kindred spirits can contribute to alleviating feelings of shame that participants may feel due to their lack of adequate digital skills. With regard to life-long learning, some participants proposed their curious personality as a reason to participate in all kinds of different courses offered to them by the public library. Most of these participants initially started on a path to improve their digital skills and this course was a natural continuation of that process to develop these further. Especially those participants who already had positive experiences with formal support at the public library were stimulated to continue with this type of support.

## Discussion

### Principal Findings

In this qualitative study, including participants of older age and with varying educational backgrounds, three motivational themes were identified for seeking help with eHealth use: (1) adapting to an increasingly digital society, (2) a sense of urgency facilitated by health care experience, and (3) a need for self-reliance and autonomy. A lack of adequate informal support from friends and family and positive experiences with nonformal support stimulated the participants to seek nonformal support in the public library for eHealth use. In addition to the 3 themes, participants expressed a more general desire for social contact with peers and lifelong learning.

The first theme “adapting to an increasingly digital society” explores the motivation to seek support driven by a wish or need for digital inclusion and societal participation in a digitalized society. It shows that the exponential development of digital technology can seem daunting and therefore insurmountable for some older adults who have little to no prior experience with this [49,50]. However, for others, it acts as a stimulus to stay engaged with societal changes, fueling the desire to adopt new technology [49,51]. In this study, this latter attitude also emerged as an important reason to seek support for eHealth use. While computer anxiety and low computer self-efficacy have been named important barriers to technology and eHealth adoption in older adults [52-54], this study shows that awareness of one’s own limited self-efficacy in eHealth use can be an important incentive to seek support when individuals see the potential usefulness for themselves as “preparation for the future.” Associated concepts such as healthy aging, aging in place (eg, living longer at home), and older person’s empowerment have all been positively linked to the use of digital (health) technology [55-58] and are reflected in the third theme.

The second theme “a sense of urgency facilitated by health care experience” revolves around participants’ prior experience with eHealth, health care, and health professionals. In this study, some participants experienced this as a necessity, while for others, this stimulated a positive attitude toward the potential benefits. Both acted as a facilitator for seeking support. Ware et al [55] equally found that a desire for ownership, access, and individual responsibility for personal medical information were motivators to use eHealth. Other research also underscores the importance of perceived eHealth benefits, positive prior eHealth experiences, and endorsement of health care professionals to motivate individual use [53,54]. This “window of opportunity” could be used to motivate others to seek nonformal support for their digital skills, for example, when introduced by trusted health professionals. However, this type of experience as a motivator for older adults to seek nonformal support for the development of digital (health) skills seems to be a novel finding.

The third theme “a need for self-reliance and autonomy” emphasizes that participants want to be self-sufficient in their abilities. This attitude toward self-reliance and autonomy illustrated how participants want to live and manage their health independently. It can support aging in place, encouraging older adults to live independently for as long as possible, a desire of most older adults and stimulated by policy makers [59,60]. Informal support by family and friends can both be a facilitator or barrier to the adoption of new digital technology [51,53,54]. In this study, part of the participants showed a dislike for dependency on family and friends and did not want to burden others turning informal support into a barrier, a sentiment also observed by others [51,53]. In this study, the help offered by family members did not lead to the development of more digital skills, as the participants desired. Therefore, they experienced limited and inadequate informal support. This particular phenomenon is seen elsewhere in informal support for internet use among older adults [61]. In addition, our findings on limited informal support are in line with others who find that intergenerational support can lack sufficient patience and understanding for the level of digital skills of older adults [51,53]. This may act as a barrier if it creates a learning environment characterized by judgmental delivery, a fast pace, and the use of unfamiliar jargon [54].

Theoretical reflection from the perspective of Maslow’s Hierarchy of Needs provides an opportunity to create a deeper understanding of the origin of the identified motivations. Maslow’s [62] theory focuses on human needs, theorizing 5 different types of needs. When these needs are not met, motivations to change behavior can emerge [62]. Furthermore, the theory implies a hierarchy of the 5 needs [62]. The basic needs are physiological needs (eg, water and food) and safety needs (eg, a roof over one’s head and the heating on) [62]. Then there are psychological needs, starting with the social need for love and belonging and moving up to the need for self-esteem [62]. Finally, there are the self-fulfillment needs, or the need for self-actualization, for example, developing one’s full potential [62]. Themes 1 and 2 both reflect a safety need. The urgency to adapt to an increasingly digital society and the urgency of being a patient or caregiver show a need for security in access to (digital) health care now or in the future. The motivations from theme 3 can be interpreted as esteem needs, as this theme can be interpreted as a need for independence in access and use of the digital world and independence from support. The motivation stemming from the desire for lifelong learning can be interpreted as a need for self-actualization. The need for social connection can be interpreted as a social need. The perspective of Maslow’s theory provides the insight that independent access to digital health care was perceived as a basic safety need and an esteem need by the participants.

Motivation to use eHealth and to seek support in eHealth use are 2 aspects of many when discussing digital health inequity. The digital divide model by van Dijk [10] describes how personal and contextual factors are related to the resources one has available to have motivation, physical access, use, and benefit from digital media, or in this case, digital health. Resources to improve the likelihood of accessing digital media involve cultural, social, mental, material, and time-related factors [10]. The study sample expressed a lack of digital skills and knowledge about digital health but also expressed access to up-to-date devices, language skills, time, and feeling comfortable in a public library setting. Our prior research and other research found that other populations experience barriers to seeking support in the factors that were facilitative for this study population [63-65]. Standaar et al [64], Choudhary and Bansal [63], and Goedhart et al [65] reported that other populations that need and potentially seek help in digital skills development experience barriers in language skills, digital skills, stigma, time, logistics, and feelings of belonging in the context of institutions. A literature review by Choudhary and Bansal [63] allowed for the categorization of the barriers to participate in training programs on the administrative level, training level, learner level, and community level. This categorization acknowledges that barriers are not only individual-bound but also bound to the training content, training context, and community in which training is provided [63]. Our findings suggest that access to up-to-date devices, language skills, time, and feeling comfortable in a public library setting were required to perform support-seeking behavior in the public library context. These reflections suggest that access to support for digital health skill development is also dependent on personal factors, contextual factors, and resources.

We explored the motivations for seeking eHealth support among participants. Additionally, we explored if differences in educational backgrounds led to differences in motivation. We found that other life events like losing connection with (digital) society, becoming a patient or caregiver, and becoming dependent on others later in life appeared to be more important in the emergence of their motivations to participate in an eHealth course. Even prior computer experience was not always an indication of the well-developed digital skills that are needed in the current digital age. Furthermore, exploration of the results through the perspective of education is complex in this older, mostly female sample as it may not clearly indicate educational differences due to historical cultural norms and educational opportunities for women in the 20th century [66]. Therefore, the results of this study provide very limited and nongeneralizable insights on different motivations to seek support in eHealth use in people from different educational backgrounds.

### Strengths and Limitations

This study has several strengths. First, participants come from diverse backgrounds, including rural and urban areas enabling exploration of a wide range of experiences. A large percentage of the potential participants (20/48, 42%) participated in the study, ensuring a broad representation of the study population. Second, interviews continued until data saturation was reached, ensuring robust findings. Third, the eHealth course was not part of a research program, which allowed us to evaluate participants’ motivations in a real-world setting. The on-site visits enhanced the understanding of the eHealth course context and allowed the interviews to take place in familiar surroundings for the participants. Our participant selection resulted in a culturally nondiverse older group with access to digital devices, motivation, and the capability to seek nonformal support, as a result of this specific research context. Next to this, it is likely that the people attending an eHealth course have some prior knowledge or experience with eHealth, the majority (n=14) of this study sample had some experience with eHealth. Other research in the same context confirms from digital health skill development trainers’ perspective that the population attending the eHealth course is homogenous and similar to this study sample [64]. Asmar et al [67] refer to this specific group as “community supported.” However, their and other research indicates that older adults are far from homogenous in their use of digital technology or support-seeking behavior [67-69]. Therefore, it is likely that in other settings, different typologies and characteristics of older adults might result in other motivations.

### Implications and Future Research

This study highlights the importance of nonformal support in using eHealth, for those lacking adequate informal support. Kebede et al [54] found that attainment of digital competence among older adults depends on awareness of existing technology and the availability of support and instruction. They stated that personalized training and opportunities for need-based learning in a safe environment facilitate skill acquisition and initial digital engagement if the learning environment is accessible and inclusive for older adults [54]. Our findings indicate that public libraries can provide this type of support for digital health skill development. The results shed light on the motivations of participants, mainly older adults, to engage in nonformal support for eHealth use, advancing digital health literacy. While existing literature has focused on facilitators and barriers of digital (health) technology adoption by older adults, measuring eHealth or digital health literacy and the effectiveness of such interventions, often in research settings [53,54,70-72], this study reveals motivations for participation in the real world. It highlights how motivations are associated with their specific needs for support and training, a need for security in access, and a desire for independent use of digital health care.

It would be interesting to explore the group of nonusers and users who do not seek nonformal support. Earlier reflections on our results suggested that this study population possessed certain characteristics, resources, and needs that align with seeking support in the public library context. Research shows that eHealth nonusers are common among other age and socioeconomic groups and that their needs for support and eHealth use are diverse [4,8,64,65,73-75]. Scholars find that populations rely on different types of support, it being formal, nonformal, and informal support, to gain access to digital skill development and eHealth [76-78]. Findings from Nygren and Hayat underline the importance of informal support to overcome digital literacy barriers. However, other findings indicate that digital inequality can manifest itself in informal support, where those who experience the most difficulties in accessing and use of digital tools are also those who have fewer resources for high-quality informal support [65,74]. Furthermore, van Deursen et al [79] show that in comparison to formal support, informal support might be insufficient to overcome all skill and content barriers for beneficial internet use. The barriers of limited access and poor quality of informal support were also found in this study, additionally, we found that discomfort with being dependent on others was also important. Smit et al [78] showed that Dutch citizens with low literacy overcome digital and literacy barriers by support from family, friends, community, and volunteers and base decisions on who they ask for support based upon the level of privacy surrounding a topic, the affective dimension involved, and the depth of knowledge possessed by the supportive actors [78]. These and our findings implicate that different populations are likely to seek support from different sources that fit their needs and personal circumstances. Further research is needed to understand the motivations, experiences, and needs in support of eHealth use by both digital technology users and nonusers. Findings can provide insights into what qualities eHealth support should entail to ensure equitable access to support and consequently to eHealth.

### Conclusions

This study indicates that participants seeking nonformal support for eHealth use were driven by a need to secure independent access and use of digital health care. This need is derived from the necessity to adapt to a digital society, the expected or current need to use digital health, and a desire to independently engage in a digital society. In general, participants were motivated to develop digital health skills they feel are necessary for societal participation. Additionally, the eHealth course met the need for social connection and lifelong learning. The study sample was a predominantly homogenous group of older female adults with language skills, access to up-to-date devices, and time. It is likely that the need for support to gain independent access and use of digital health care is present in other populations. Future research should explore the needs and attitudes of users and nonusers of digital health toward support in eHealth access.

## Supplementary material

10.2196/60612 Multimedia Appendix 1 Interview guide used to conduct semistructured face-to-face interviews with participants of the eHealth course DigiVitaler in the context of Dutch public libraries about their motivations to seek support in eHealth use.

10.2196/60612 Multimedia Appendix 2 Demographics of the study participants interviewed between April and June 2022. Participants were categorized in educational level based upon the international standard classification of education level (ISCED). Rurality of the public library the participants attended the eHealth course was determined based upon zip code of the library and the indexed rurality by Netherlands Statistics.

10.2196/60612 Multimedia Appendix 3 Overview of inductive themes and inductive topics and number of codes per topic derived from twenty interviews that were conducted with participants from an eHealth course in Dutch public library settings.

10.2196/60612 Checklist 1 Standards for Reporting Qualitative Research checklist

## Abbreviations

ISCED: International Standard Classification of Education
SRQR: Standards for Reporting Qualitative Research
