# Supplementary material for: The Motivations of Citizens to Attend an eHealth Course in the Public Library: Qualitative Interview Study
Source: JMIR Form Res. 2025 Apr 28;9:e60612. doi: 10.2196/60612 (PMC12052220; doi:10.2196/60612)
Supplement: Multimedia Appendix 1 [file formative-v9-e60612-s001.docx]

**Introduction (2 minutes)**

First of all, thank you for agreeing to participate in this conversation.

Introduction to Digital Care

The world is becoming increasingly online and digital. Through mobile phones, computers, and the internet, we can manage more and more things. This also applies to your health. Care delivered via a computer or mobile phone is called digital care. Digital care is used to provide services, such as assisting with diagnoses or supporting therapies.

In public health, digital care is used to share information about health and healthy lifestyles via websites or mobile applications. It is also used to help with administrative tasks, like booking appointments or requesting repeat prescriptions online.

Many people need assistance using digital care. The government wants to learn more about experiences with support for digital care—what is working well and what could be improved. The ideas of participants in this course are very important! In this conversation, we will discuss:

Your opinions about digital care

Support for using digital care

This conversation will take about an hour.

**Consent to Recording (1 minute)**

Go over the consent form and sign it.

Before we start, may I ask if I can record this conversation? The purpose of the recording is to review your ideas and opinions afterward. The recording and the results of the discussions will not be traceable to you. The recording will be stored securely on our computer, accessible only to the research team. Your name will not appear on the recording, which will be assigned a number instead. Recordings will never be made public and will be deleted after the study concludes.

Would you like to participate in the study? If so, could you please sign this form?

Do you agree to let me record the conversation?

If yes: Start the recording.

**Ground Rules (1 minute)**

We are interested in your experiences and ideas, so there are no right or wrong answers. If you don’t wish to answer a question, feel free to let us know, and we will skip it.

Do you have any questions before we begin?

**Introductions (5 minutes)**

Before we start, let me introduce myself. My name is Lucille, and I live in The Hague. I am conducting research on how best to help people use digital care.

Would you like to introduce yourself? Where do you live, and what do you do during the day?

What year were you born? / Are you above or below 50 years old?

What is the highest level of education you’ve completed?

What kind of work do you do/did you do?

Do you have a smartphone (with internet)? Do you have a computer at home (with internet)? What do you use them for?

How is your health?

If someone else is present during the interview, please also note their presence.

**The Interview (45 minutes)**

General Help Questions (for interviewer only)

Can you tell us more about it?

Can you give an example?

That’s interesting—what happened then?

How was that for you?

You mentioned …; what do you mean by that?

**Main Themes**

Starting Question: How did you come across this course?

**Experience with E-Health**

Have you used digital care before taking this course? Examples include websites like thuisarts.nl, step counters, repeat prescriptions, GP websites, or online appointments.

If yes: Can you tell us more about it? (Reason, format, experience—positive/negative, usefulness)

If no: Have you ever tried using digital care?

**Introduction to E-Health**

Why did you start using digital care?

Was it your initiative or encouraged by family, friends, or healthcare providers?

Experience with Support (Libraries and Others)

What do you think of the course so far?

Do you feel confident using digital care at home after this course?

If yes: What about the course helped you?

If no: What is still missing?

**Support from Others**

Do you receive help from others with digital care? (Family, friends, caregivers)

If yes: How did they support you? Was it enough?

If no: Would you have liked support?

**Closing Question**

This report will be sent to the Ministry of Health. Digital care is increasingly being seen as a solution in healthcare. What would you like to share with the Ministry to improve digital care?

**Closing Questions (3 minutes)**

Are there any other points you’d like to share?

Do you feel like you’ve been able to say everything on this topic?

**Conclusion (5 minutes)**

This concludes our conversation. Would you like to add anything else?

What did you think of the interview?

Thank you again for your time and effort. We will process this and other interviews into a report, which will be presented to the government. Would you like to receive a copy of the report?

Once again, thank you for your time and effort!

**Compensation**

Provide a gift card (VVV-bon) and leave a phone number for any follow-up questions.
