# Supplementary material for: The Motivations of Citizens to Attend an eHealth Course in the Public Library: Qualitative Interview Study
Source: JMIR Form Res. 2025 Apr 28;9:e60612. doi: 10.2196/60612 (PMC12052220; doi:10.2196/60612)
Supplement: Multimedia Appendix 2 [file formative-v9-e60612-s002.docx]

| Overview of participants | | | | | | | |
| --- | --- | --- | --- | --- | --- | --- | --- |
| No. interview | Sexe, (F/M)^[[1]](#footnote-1)^ | Age (years) | Educational level (ISCED #)^[[2]](#footnote-2)^ | Ethnic background | Location of public library (rural/urban) | Use of digital devices (type of device) | Prior (to the interview) experience digital health services (yes/no: what kind) |
| 1 | F | 73 | Short-cycle tertiary education (ISCED 5) | White | Rural-urban | Tablet, laptop | Yes: patient portal, health information websites |
| 2 | M | 79 | Post-secondary non-tertiary education (ISCED 4) | White | Rural-urban | Smartphone, desktop computer | Yes: refill medication prescription |
| 3 | F | 78 | Master’s or equivalent level (ISCED 7) | White | Urban | Smartphone, laptop | Yes: health information websites |
| 4 | F | 82 | Lower secondary education (ISCED 2) | White | Urban | Smartphone, tablet, laptop | No |
| 5 | F | 72 | Lower secondary education (ISCED 2) | White | Urban | Smartphone, laptop | Yes: patient portal, doctor appointments |
| 6 | F | 66 | Lower secondary education (ISCED 2) | White | Rural-urban | Smartphone, laptop | No |
| 7 | F | 72 | Upper secondary education (ISCED 3) | White | Urban | Smartphone, tablet, laptop | Yes: health information websites, doctor appointments, refill medication prescription |
| 8 | F | 76 | Lower secondary education (ISCED 2) | White | Urban | Smartphone, laptop | No |
| 9 | M | 70 | Upper secondary education (ISCED 3) | White | Urban | Smartphone, desktop computer | Yes: health information websites |
| 10 | F | 78 | Short-cycle tertiary education (ISCED 5) | White | Urban | Smartphone, laptop, desktop computer | No |
| 11 | F | 73 | Bachelor’s or equivalent (ISCED 6) | White | Rural | Smartphone, tablet, desktop computer | Yes: patient portal, health information websites |
| 12 | F | 80 | Upper secondary education (ISCED 3) | White | Rural | Smartphone, tablet, desktop computer | Yes: health information websites, health apps |
| 13 | F | 70 | Primary education (ISCED 1) | White | Rural-urban | Smartphone, tablet, desktop computer, smartwatch | Yes: patient portal, health apps |
| 14 | F | 76 | Upper secondary education (ISCED 3) | White | Rural-urban | Smartphone, desktop computer | Yes: patient portal |
| 15 | F | 74 | Bachelor’s or equivalent level (ISCED 6) | White | Urban | Smartphone, desktop computer | Yes: health information websites |
| 16 | M | 79 | Upper secondary education (ISCED 3) | White | Urban | Smartphone, desktop computer | No |
| 17 | F | 51 | Bachelor’s or equivalent level (ISCED 6) | White | Urban | Smartphone, laptop | Yes: health apps |
| 18 | F | 76 | Lower secondary education (ISCED 2) | White | Urban | Smartphone, tablet, laptop, smartwatch | Yes: health information websites |
| 19 | F | 74 | Lower secondary education (ISCED 2) | White | Urban | Tablet, desktop computer, smartwatch | No |
| 20 | M | 73 | Master’s or equivalent (ISCED 7) | White | Urban | Smartphone, tablet, desktop computer, smartwatch | Yes: patient portal |

1. F: Female, M: Male [↑](#footnote-ref-1)
2. ISCED: International standard classification of education [↑](#footnote-ref-2)
