# Supplementary material for: The Motivations of Citizens to Attend an eHealth Course in the Public Library: Qualitative Interview Study
Source: JMIR Form Res. 2025 Apr 28;9:e60612. doi: 10.2196/60612 (PMC12052220; doi:10.2196/60612)
Supplement: Multimedia Appendix 3 [file formative-v9-e60612-s003.docx]

| **Theme 1 – Adapting to an increasingly digital society** | | **Number of codes** |
| --- | --- | --- |
|  | I have to keep up with the digitalizing world in order to stay part of society | 7 |
|  | I need help with the development of my digital skills | 14 |
|  | I have to prepare myself for a future where e-health is the norm | 7 |
|  | I want to prepare myself for a future where e-health is the norm | 6 |
|  | I see the benefits and the necessity of e-health | 10 |
| **Theme 2 – Sense of urgency facilitated by experience with health care** | |  |
|  | I'm a patient or a caregiver and I need to know how to use e-health | 5 |
|  | I was exposed to e-health | 3 |
|  | I see the benefits of e-health and I am eager to use it | 2 |
|  | I'm a patient or a caregiver | 8 |
|  | I see the benefits and the necessity of e-health | 10 |
| **Theme 3 – Need for self-reliance and autonomy** | |  |
|  | My social network can't provide adequate help | 5 |
|  | I want to be independent from others | 5 |
|  | I want to be self-sufficient in using e-health | 3 |
|  | My social network can't provide adequate help | 3 |
|  | I want to determine how I receive care and how I get access to health care information | 4 |
